# Occupational health check-ups and health-promoting programs and asthma

**DOI:** 10.1186/s12889-020-09403-z

**Published:** 2020-08-31

**Authors:** Riina Hakola, Timo Leino, Ritva Luukkonen, Paula Kauppi

**Affiliations:** 1 Department of Public Health, University of Helsinki, PO Box 40, 00014 Helsinki, Finland; 2 The Finnish Institute of Occupational Health, Helsinki, Finland; 3 Skin and Allergy Hospital, Helsinki University Hospital, Helsinki, Finland

**Keywords:** Asthma, Depression, Health-promoting/behavior, Obesity, Smoking, Physical workload

## Abstract

**Background:**

The focus in occupational health check-ups is in work and health, but they offer also a possibility to assess health behavior and give guidance e.g. on weight control. We wanted to study whether having occupational health checks-up, receiving physicians’ advice to change health behavior or participation in health promotion programs had an effect on obesity in a five-year follow-up from 1998 to 2003 in asthmatic and non-asthmatic workers.

**Methods:**

Altogether 23,220 individuals aged 20–54 years were picked up from a randomized Finnish population sample. Univariate and multivariate logistic regression analysis was used to calculate the risk for obesity in 2003. The variables used in the modelling were gender, age, smoking, asthma, depression, and physical workload.

**Results:**

Both asthmatic and non-asthmatic workers gained weight during the follow-up. Of the asthmatics 48 and 47% of the non-asthmatics had occupational health-check-up in the last 5 years. Of the asthmatics 18 and 14% of the non-asthmatics had received physician’s advice to change their health behavior (*p* < 0.001). Associated factors for obesity (BMI > 30) in 2003 were gender (men OR 1.19), older age (OR 1.25), smoking (OR 1.07) or depression (OR 1.44).

**Conclusions:**

Results show that having occupational health checks-up or receiving physicians’ advice to change health behavior or participation in health promotion programs did not stop gain of weight during a five-year follow-up. Asthmatic workers did not differ from non-asthmatics. Male gender, older age, smoking, and depression were associated with obesity but not the physical workload.

## Background

Finland has universal social security scheme. Preventive services are provided by health centers, child health clinics, school health services, student health care and occupational health services. Employers are responsible for providing employees with preventive health care and voluntarily also medical care. The Finnish working-age population (2.6 million) is under health surveillance for both public health and occupational health purposes. The occupational health service carries out specific health examinations of the working population divided into pre-employment, special examinations for workers in hazardous jobs, when returning to work after a long sick leave, for the assessment of work-ability, and after retirement from certain hazardous jobs according to the Act on Occupational Health Services. Annually approximately 1 million health examinations are done in occupational health services [1]. The focus in examinations is in work and health, but also health behavior and assessment of lifestyle risk factors are part of check-ups. Although evidence on check-ups’ effect on morbidity or mortality is scanty [2, 3], guidance to maintain promote health and workability is seen important in the Finnish occupational health service.

Asthma is the most common chronic disease affecting the working aged population of all ages and in Finland the prevalence of asthma is 9.6% although occupational asthma is diagnosed only in 5% of all asthma cases. Likewise, the prevalence of chronic obstructive lung disease (COPD) is approximately 6% in Finland and globally it is the third most common cause of death and the fifth most common cause common cause of work disability and overall disability although it is rarely an occupational disease [4–6].

In a general Finnish population, smoking has been decreasing and obesity increasing [7]. Although the trends of health behavior have been studied in a general population, far less is known whether health behavior has changed in asthma patients in Finland in the last years. Both smoking and obesity are associated with asthma which overlaps with COPD [8]. Both lung diseases are important when considering work-ability [9].

The focus in occupational health check-ups in work and health, but it also offers a possibility to assess health behavior and give guidance e.g. on weight control. This study will give new information, whether having occupational health check-ups or receiving physicians’ advice to change health behavior or participation on health-promoting programs had an impact on health behavior (determined as obesity) in asthmatic and non-asthmatic workers in the follow-up time from 1998 to 2003.

## Methods

The Health and Social Support (HeSSup) Study is prospective and included altogether 23,879 individuals. The cohort is a random population sample of the Finnish population aged 20–54 years followed by surveys conducted in 1998 and 2003 [9, 10]. We picked up 23,220 individuals aged 20–54 years in 1998. Information on physician-diagnosed asthma and work status were driven from self-reported questionnaire data. Asthma was defined as self-reported physician-diagnosed asthma in 1998 when chronic bronchitis was excluded.

Age was analysed as using the following groups: 20–24, 30–34, 40–44, and 50–54 years. Weight was analysed in two body mass index (BMI) categories: < 30 normal or minor overweight (BMI 18–29.9) and obesity (BMI > 30) in 1998 and 2003. Smoking habits were assessed as having never smoked (non-smoker), previously (ex-smoker), or currently (current smoker) smoking regularly in 1998 and 2003. Beck depression scale [11] was used to analyze depression symptoms (< 10 = no depression, healthy and > 10 = depressed) [10]. The physical workload was categorized as very light (sitting), light (mostly sitting and standing), moderate (standing and walking), and heavy (walking, lifting, and carrying). Use of health care services was assessed by the questions ‘Have you had an occupational health check-up?’ (none, in the last year, 1–5 years ago, more than 5 years ago), ‘Have you participated in health-promoting programs such as smoking cessation or weight loss program?’ (none, in the last year, 1–5 years ago, more than 5 years ago). ‘Has your physician advised you to change your health behavior?’ (no, yes).

The Chi-square test was used to analyse differences in the frequency distribution of categorical variables and Student’s t-test of continuous normally distributed variables. Univariate and multivariate logistic regression analysis was used to calculate the risk for obesity in 2003 with 95% confidence intervals (CI). The variables used in the modelling were gender, age, smoking, asthma, depression and physical workload. Spearman’s correlation coefficient was calculated for correlation of occupational health check-ups and obesity in 2003. Multivariate logistic regression analysis was done using gender, age, smoking, asthma status, depression, and physical workload. Analyses were performed with IBM SPSS Statistics software (version 19.0) (IBM Corporate, New York, USA). The study was approved by the Ethical Committee of Turku University Central Hospital.

## Results

### Participation rate

In the year 1998 altogether 25,901 responded to the questionnaire study with a response rate of 40.0% [12]. In the follow-up 5 years later, the response rate was 80.2%. Of the respondents in the year 1998 altogether 23,220 study individuals were included in this study. Drop out of the original whole study population in here between the years 1998 and 2003 was 3830. Those who did not answer the questionnaire in 2003 were more often men (53%) but did not differ in respect to obesity (10% obese) or to smoking status (28% current smokers).

In 1998, of all the respondents 60.7% had had an occupational health check-up. In 1998, altogether 1310 (6.1%) participants had participated in a health-promoting program earlier.

### Prevalence of self-reported obesity

According to self-reported information, prevalence of obesity (BMI > 30) was 9.1% (*n* = 2105) in year 1998 and 13.0% (*n* = 2264) in year 2003. There were more obese individuals (BMI > 30) among asthmatics (12%) than non-asthmatics (9%) in 1998 (p 0.014). In the five-year follow-up time, obesity increased and at the end of the follow-up 16% of the asthmatics and 13% of the non-asthmatics were obese (p 0.05). The mean BMI of the non-asthmatics was 25 (SD 4.1) in 1998 and 25 (SD 4.5) in 2003 (*p* < 0.001). The greatest change in BMI was in those not having had an occupational health-check-up (+ 0.9, SD 2.6). Respectively, those reporting participation in health-promoting program during the last 1–5 years in 1998 gained the most weight during the follow-up (+ 1.1, SD 3.5).

### Prevalence of asthma and other health-related outcomes

**Fig. 1:**
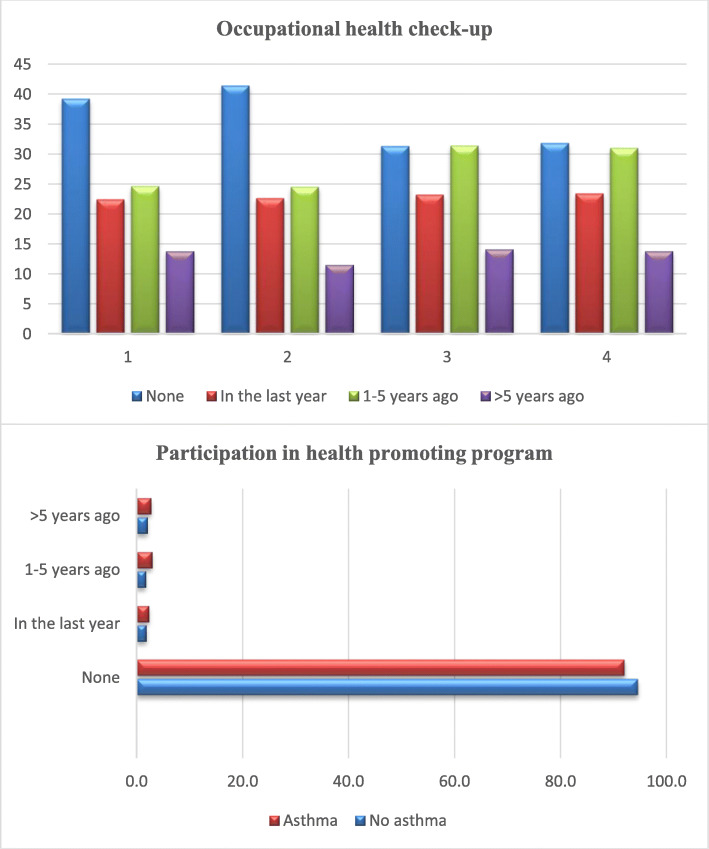
Participation in occupational health check-up (1) no asthma, in 1998 (2) asthma, in 1998 (3) no asthma, in 2003 (4) asthma, in 2003 (upper panel). Participation in health promoting program participation in 2003 (lower panel)

The prevalence of self-reported asthma was 3.7% (*n* = 848) in the total study population in the year 1998. Asthmatics were more often women (62%) and they were younger (35% aged 20–24 years) than non-asthmatics (58%, p 0.021 and 28% aged 20–24 years, *p* < 0.001, respectively) (Table 1). At the beginning of the follow-up, 48% of asthmatic participants reported having had an occupational health check-up in the last 5 years when the respective number for the non-asthmatics was 47% (Table 1, Fig. 1). Physical workload, smoking habits, percentage of depressed individuals, and participation in occupational health check-ups were similar in asthmatics and non-asthmatics in 1998 when the follow-up began. Physician’s advice to change health behavior was given more often to asthmatics (18%) than non-asthmatics (14%) (*p* < 0.001).

**Table 1:** Characteristics of the study population in 1998, asthma defined as self-reported physician-diagnosed asthma ever (chronic bronchitis excluded). BMI (body mass index), Beck depression scale and health-promoting program participation presented also in 2003

	No asthma 1998	%	Asthma 1998	%	Total	***P***-value
**Gender**						
Women	13,064	58	529	62	13,593	
Men	9308	42	319	38	9627	
All	22,372	100	848	100	23,220	0.021
**Physical work load 1998**						
Very light	7160	42	290	46	7450	
Light	2445	14	90	14	2535	
Moderate	6058	35	203	32	6261	
Heavy	1543	9	54	9	1597	0.224
**Occupational health check-up 1998**						
None	8706	40	348	41	9054	
In the last year	4976	22	190	23	5166	
1–5 years ago	5462	25	206	25	5668	
More than 5 years ago	3052	14	97	12	3149	0.274
**Age group 1998**						
20–24	6203	28	296	35	6499	
30–34	5366	24	240	28	5606	
40–44	5364	24	155	18	5519	
50–54	5438	24	157	19	5595	< 0.001
**Has your physician advised you to change your health behavior? 1998**						
No	19,090	87	685	82	19,775	
Yes	2982	14	153	18	3135	< 0.001
**Have you participated in a health-promoting program? 1998**						
None	20,844	94	776	93	21,620	
In the last year	472	2	24	3	496	
1–5 years ago	425	2	21	3	446	
More than 5 years ago	353	2	15	2	368	0.280
**Smoking 1998**						
Non-smoker	9504	46	359	46	9863	
Ex-smoker	5689	28	222	29	5911	
Current smoker	5360	26	194	25	5554	0.753
**Smoking 2003**						
Non-smoker	7533	49	290	50	7823	
Ex-smoker	4674	30	171	30	4845	
Current smoker	3328	21	117	20	3445	0.693
**BMI 1998**						
< 30	20,240	91	746	89	20,986	
≥ 30	2008	9	97	12	2105	0.014
**BMI 2003**						
< 30	14,676	87	533	85	15,209	
≥ 30	2166	13	98	16	2264	0.050
**Beck depression scale 1998**						
Healthy (0–9)	19,834	89	747	89	20,581	
Depressed (≥10)	2378	11	94	11	2472	0.665
**Beck depression scale 2003**						
Healthy (0–9)	14,725	89	542	86	15,267	
Depressed (≥10)	1894	11	85	12	1979	0.096
**Have you participated in a health-promoting program? 2003**						
None	15,760	95	571	92	16,331	
In the last year	299	2	14	2	313	
1–5 years ago	278	2	18	3	296	
More than 5 years ago	325	2	17	3	342	0.040

In the five-year follow-up time smoking decreased in both the groups of asthmatics and non-asthmatics.

In 1998, 4.0% of the non-asthmatics (2.1% in the last year and 1.9% 1–5 years ago) and 5.4% of the asthmatics (2.9% in the last year and 2.5% 1–5 years ago) reported participation in a health- promotion program on smoking cessation or weight-loss in the last 5 years. In 2003, 3.5% of the non-asthmatics and 5.1% of the asthmatics reported participation in a health promotion program in the last 5 years (Table 1, Fig. 1).

Further, obese working-aged individuals were advised almost three times more often to change their health behavior (11% vs 30% in 19,998, *p* < 0.001). Obese working aged individuals participated 1.5 times more often in the weight-loss program (7% in the last year, 5% in the last 1–5 years and 4% more than 5 years ago vs 5% in the last years, 5% in the last 1–5 years, and 3% more than 5 years ago) (p < 0.001) than the those with normal weight or mild overweight.

### Univariate and multivariate models

Asthma in the year 1998 increased the risk of obesity in univariate analysis (OR 1.27). However, when other factors were taken into model, asthma was not any more a significant risk factor for obesity. Associated factors for obesity (BMI > 30) in 2003 were gender (men OR 1.19), older age (OR 1.19), and mild and moderate to severe depression (OR 1.44) (Table 2). In addition, smoking increased the risk of obesity in 2003 (OR 1.08) statistically significantly.

**Table 2:** Logistic regression (univariate and multivariate) for obesity (BMI > =30) in 2003

Logistic regression for obesity (BMI > =30 in 2003)								
		n	OR	95%CI	OR	95%CI	P (univariate)	P (multivariate)
**Asthma 1998**	No	16,485	1.0		1.0			
	Yes	612	**1.27**	**1.02–1.58**	1.26	0.95–1.65	0.035	0.106
**Gender 1998**	Women	10,410	1.0		1.0			
	Men	6712	**1.15**	**1.05–1.26**	**1.19**	**1.07–1.33**	0.020	0.002
**Physical work load 1998**	Very light	5690	1.0		1.0		0.029	
	Light	1965	0.89	0.76–1.05			0.163	
	Moderate	4694	1.04	0.93–1.16			0.540	
	Heavy	1076	**1.23**	**1.03–1.48**	1.04	0.99–1.09	0.024	0.153
**Age group 1998**	20–24	4447	1.0		1.0		< 0.001	
	30–34	3965	**1.36**	**1.18–1.57**			< 0.001	
	40–44	4216	**1.65**	**1.44–1.89**			< 0.001	
	50–54	4494	**2.27**	**2.27–2.58**	**1.25**	**1.19–1.32**	< 0.001	< 0.001
**Smoking 1998**	Non-smoker	7549	1.0		1.0			
	Ex-smoker	4376	**1.32**	**1.18–1.47**			< 0.001	
	Current smoker	3811	**1.21**	**1.08–1.36**	**1.08**	**1.01–1.15**	0.001	0.033
**Beck depression**	Healthy (0–9)	13,991	1.0		1.0		< 0.001	
**scale 1998**	Mild depression (10–17)	2252	1.58	**1.40–1.78**			< 0.001	
	Moderate depression (18 or more)	763	2.57	**2.17–3.05**	**1.44**	**1.30–1.58**	< 0.001	< 0.001

## Discussion

Our results show that having occupational health checks-up or receiving physicians’ advice to change health behavior or participation in health promotion programs did not stop weight gain during a five-year follow-up. Asthmatic workers did not differ from non-asthmatics. Male gender, older age, smoking and depression were associated with obesity.

Participation in the occupational health check-ups was good, since 54 and 55% of the working asthmatics and non-asthmatics had had an occupational health check-up in the last 5 years or earlier. Physician’s advice had been well focused on asthmatics, since they had received advice to change their health behavior significantly more often compared to the non-asthmatic workers.

Despite having received more often advice to change health behavior (18% of the asthmatics and 14% of the non-asthmatics, *p* < 001) current smoking was as frequent and obesity even more frequent among asthmatic than non-asthmatic workers.

Participation in health-promoting programs such as in a weight-loss program was rare. The greatest weight gain was observed in those reporting participated in a health-promoting program during the last 1–5 years in 1998 and in those who reported participation in a program in the last year in 2003. The change of BMI was most significant in those reporting not having an occupational health check-up neither in 1998 nor in 2003. Whether those with greatest weight gain were offered a possibility for an occupational health check-up cannot be estimated with this data. Male gender, older age, smoking, and depression were risk factors for obesity in 2003. In generalized linear model only weight in 1998 and depression was predictive for obesity in 2003. According to these results, participation in health-promoting programs was advised correctly but participation was low. The occupational health check-ups did not achieve those with a significant increase in BMI at the 5-year follow-up. Elsewhere, it has been suggested to target the promotion of healthy diet to boys and men and people with low education [13].

Occupational health-check-ups (in 1998) correlated with obesity in 2003 (r 0.027, p 0.001) which probably reflects targeted interventions but without entire success to prevent obesity. Instead of being single acts or a short intervention occupational health check-up are a long- term process with set targets, follow-up, and assessment. In addition, occupational health nurses are mostly (2/3) conducting the health check-ups in Finland [1], and by the nature of their education better equipped for health promotion than physicians in general. Further, obese working-aged individuals were adviced almost three times more often to change their health behavior and participated 1.5 times more often in health-promoting programs such as smoking cessation and weight-loss program which also reflects the number of given interventions. In a study of New Zealand, it was also found that current health care interventional programs are not effective enough in means of reducing obesity and that new and broader perspectives should be encouraged if positive results were expected [14]. Contrarily, implementing health promotion programs has been suggested to reduce health-care costs by employees with high blood glucose, obesity, stress, depression, and physical inactivity [15].

Weight control in asthma is important since an increase in the severity of asthma is associated with an increase in obesity [16]. Controversially, also miss-diagnosis of asthma in obese individuals has been reported [17]. In the study of Mosen el al. obese individuals with persistent asthma were significantly more likely than those with normal BMIs to report worse asthma-related quality of life and more asthma-related hospitalizations [18]. In their study, obesity was an independent risk for hospitalization even after adjustments for age, gender, smoking status, use of corticosteroids, and gastroesophageal reflux disease. Here, obesity was associated with asthma only at the beginning of the follow-up (1998) but did not remain as an independent risk factor of obesity in 2003. Also, asthmatic workers were younger than non-asthmatics and obesity was associated with older age in the follow-up. Further, depression increased the risk of obesity in 2003. While obesity has a considerable amount of negative side effects, however, it has been reported to be protective for mortality in COPD [19, 20].

Moderate to heavy workload is usually associated with lower educational levels and work status (blue-collar work). In other studies, a higher prevalence of current smoking has been associated with lower educational level and a more appropriate focusing of smoking cessation and other tobacco control policies has been suggested [20, 21]. Our results are on agreement with the previous ones with current smoking concentrating in the groups with moderate to heavy physical workload (data not shown). Written information, brief interventions, and group counseling can be used in smoking cessation. 18% of the asthmatic workers and 14% of the non-asthmatic workers had received advice to change health behavior when the follow-up begins but only 5 and 4% of the working-aged individuals had participated in health promotion program in the last 5 years according to their responses in 2003. Elsewhere, both brief interventions and written information have been reported to be effective in the occupational health care setting in reducing hazardous alcohol consumption [22].

In addition, individual counseling, group therapy, and nicotine replacement therapy have been effective in smoking cessation at the workplace [23]. Here, the percentage of obese individuals increased in both the non-asthmatic and asthmatic groups in the follow-up period. Effective health promotion strategies in adolescents have been reported to focus on increasing self-esteem and self -empowerment rather than on single health issues and that interventions should be simultaneous at government, community, and the local level [24]. In the same paper, it was pin-pointed that health risks clustered and that those who were regular smokers had also more often regular consumption of alcohol [24]. Recent results suggest that mobile apps might bring a practical tool for diet and physical activity interventions by monitoring health status and behavior change and by providing feedback [25].

Mobile apps have been used not only to diet and activity advise, but also to overall healthy lifestyle improvement. The apps should be designed not only to monitor health behavior and behavior change but also to provide feedback and thus to aid and improve behavior change. In general, using apps has been reported to be more effective in health behavior change when compared to those not using apps [25]. Further, increasing the physical activity of overweight individuals into the same level of normal weight individuals leads to a reduction in risk of work disability by 3–20% regardless of the possible change in weight [26].

The strengths of this study are the population base study design which offers an unbiased sample of the working population and the prospective study design [27]. Limitation and weaknesses of the study is a relatively short period for the follow-up (5 years) and lack of detailed information on the content of the health promotion program. By formulating the questions to be more specific it would have been possible to specify in what kind of health promotion program the patient had participated.

Since this is a questionnaire survey, study participants may have participated in a health promotion program even without having had an occupational health check-up. These health promotion programs may have organized also in primary health care. Further, since this is a questionnaire survey, we have not weighted the study individuals and used self-reported information on weight. This might have resulted in a reporting bias (reporting better figures in weight than if measured) and selection bias (those responded in the questionnaire who were less obese).

Although asthmatics received more often than non-asthmatics advice to change health behavior, participation in health promotion programs, such as weight control programs was low and results poor. No effect on obesity was found. Obesity in 1998 predicted obesity in 2003. Asthmatics did not differ from non-asthmatics during a five-year follow-up time; both gained weight. Male gender, older age, smoking, and depression were associated with obesity. More attention and future research should be paid to the use of motivational counseling and empowerment techniques, the long-term support, and the follow-up of individuals gaining weight, since obesity is a known risk factor for both asthma and severe asthma.

The focus in occupational examinations is in work and health, but also health behavior and assessment of lifestyle risk factors and health-promoting advice are given when necessary. However, information on the effect of physician’s guidance or health-promoting programs on health behavior changes is limited. We report results on epidemiologic data. Here, asthmatic workers received more often than non-asthmatic workers physician’s advice to change health behavior and participated more often in health promotion programs. Male gender, older age, smoking, and depression were associated with obesity. Previous weight and depression predicted future obesity and change in BMI was especially significant in those not participating in occupational health check-ups.

## Conclusions

Our results show that having occupational health checks-up or receiving physicians’ advice to change health behaviour or participation in health promotion programs did not stop weight gain during a five-year follow-up. Asthmatic workers did not differ from non-asthmatics from the perspective that asthma as a disease was not a risk factor for obesity at the end of follow-up when adjusting for other factors. Male gender, older age, smoking, and depression were associated with obesity.

## Data Availability

Data and material can be requested from TL.

## Abbreviations

BMI: Body mass index
OR: Odds ratio
COPD: Chronic obstructive pulmonary disease
HeSSup: The health and social support study
CI: Confidence intervals
SD: Standard deviation

## References

[1] Social Insurance Institution’s Annual statistics on occupational health services. Kela, Helsinki; 2010. http://www.kela.fi. Accessed 6 July 2014.

[2] Krogsboll L, Jorgensen K, Larsen C, Gotzsche P. General health checks in adults for reducing morbidity and mortality from disease: Cochrane systematic review and meta-analysis. BMJ. 2012;345:e7191. 10.1136/bmj.e7191 PMC3502745 23169868

[3] Mahmud N, Schonstein E, Schaafsma F (2010). Pre-employment examinations for preventing occupational injurya and disease in workers. Cochrane Database Syst Rev.

[4] Kainu A, Rouhos A, Sovijärvi A, Lindqvist A, Sarna S, Lundbäck B. COPD in Helsinki, Finland: socioeconomic status based on occupation has an important impact on prevalence. Scand. J. Public Health. 2013;41:570–8. 10.1177/1403494813484554 23599377

[5] Lozano R, Naghavi M, Foreman K, Lim S, Shibuya K, Aboyans V (2012). Global and regional mortality from 235 causes of death for 20 age groups in 1990 and 2010: a systematic analysis for the global burden of disease study 2010. Lancet..

[6] Vos T, Flaxman AD, Naghavi M, Lozano R, Michaud C, Ezzati M (2012). Years lived with disability (YLDs) for 1160 sequelae of 289 diseases and injuries 1990-2010: a systematic analysis for the global burden of disease study 2010. Lancet..

[7] Murto J, Kaikkonen R, Pentala-Nikulainen O, Koskela T, Virtala E, Härkänen T, Koskenniemi T, Jussmäki T, Vartiainen E & Koskinen S. Aikuisten terveys-, hyvinvointi- ja palvelututkimus ATH:n perustulokset 2010-2017. In Finnish. (Report on Health, welfare and health care use in adults). www.thl.fi/ath Accessed 31 May 2014.

[8] Kauppi P, Kupiainen H, Lindqvist A, Tammilehto L, Kilpeläinen M, Kinnula VL (2011). Overlap syndrome of asthma and COPD predicts low quality of life. J Asthma.

[9] Hakola R, Kauppi P, Leino T, Ojajärvi A, Pentti J, Oksanen T (2011). Persistent asthma, comorbid conditions and the risk of work disability: a prospective cohort study. Allergy..

[10] Sumanen M, Koskenvuo M, Immonen-Räihä P, Suominen S, Sundell J, Mattila K (2004). Secondary prevention of coronary heart disease is disappointing among patients of working age. Fam Pract.

[11] Beck AT, Ward CH, Mendelson M, Mock J, Erbaugh J (1961). An inventory for measuring depression. Arch Gen Psychiatry.

[12] Virtanen P, Vahtera J, Broms U, Sillanmäki L, Kivimäki M, Koskenvuo M (2008). Employment trajectory as determinant of change in health-related lifestyle: the prospective HeSSup study. Eur J Pub Health.

[13] Mader S, Rubach M, Schaecke W, Röger C, Feldhoffer I, Thalmeier EM (2020). Healthy nutrition in Germany: a survey analysis of social causes, obesity and socioeconomic status. Public Health Nutr.

[14] Mandlik M, Oetzel JG, Kadirov D. Obesity and health care interventions: substantiating a multi-modal challenge through the lens of grounded theory. Health Promot J Austr. 2020. 10.1002/hpja.347. 10.1002/hpja.347 32304614

[15] Goetzel RZ, Henke RM, Head MA, Benevent R, Rhee K (2020). Ten modifiable health risk factors and Employees’ medical costs-an update. Am J Health Promot.

[16] Browatzki A, Ulrik CS, Lange P (2009). Prevalence and severity of self-reported asthma in young adults. Eur Respir J.

[17] Scott S, Currie J, Albert P, Calverley P, Wilding JP (2012). Risk of misdiagnosis, health-related quality of life, and BMI in patients who are overweight with doctor-diagnosed asthma. Chest.

[18] Mosen DN, Schatz M, Magid DJ, Camargo CA (2008). The relationship between obesity and asthma severity and control in adults. J Allergy Clin Immunol.

[19] Cazzola M, Calzetta L, Lauro D, Bettoncelli G, Cricelli C, Di Daniele N, et al. Asthma and COPD in and Italian adult population: Role of BMI considering the smoking habit. Respir. Med. 2013;107(9):1417–22. 10.1016/j.rmed.2013.04.021 23702090

[20] Cao C, Wang R, Wang J, Bunjhoo H, Xu Y, Xiong W (2012). Body mass index and mortality in chronic obstructive pulmonary disease: a meta-analysis. PLoS One.

[21] Gorini G, Carreras G, Allara E, Faggiano F (2013). Decennial trends of social differences in smoking habits in Italy: a 30-year update. Cancer Causes Control.

[22] Asch DA, Muller RW, Volpp KG (2013). Conflicts and compromises in not hiring smokers. N Engl J Med.

[23] Cahill K, Moher M, Lancaster T (2008). Workplace interventions for smoking cessation. Cochrane Database Syst Rev.

[24] Viner R, Macfarlane A (2005). Health promotion. BMJ..

[25] Lee M, Lee H, Kim Y (2018). Mobile App-Based Health Promotion Programs: A Systematic Review of the Literature. Int J Environ Res Public Health.

[26] Ervasti J, Airaksinen J, Pentti J (2019). Does increasing physical activity reduce the excess risk of work disability among overweight individuals?. Scand J Work Environ Health.

[27] Korkeila K, Suominen S, Ahvenainen J (2001). Non-response and related factors in a nation-wide health survey. Eur J Epidemiol.

